# On multivariate imputation and forecasting of decadal wind speed missing data

**DOI:** 10.1186/s40064-014-0774-9

**Published:** 2015-01-13

**Authors:** Ronald Wesonga

**Affiliations:** School of Statistics and Planning, Makerere University, P.O. Box 7062, Kampala, Uganda

**Keywords:** Wind speed, Missing data, Imputations, Forecasting, Statistical models

## Abstract

This paper demonstrates the application of multiple imputations by chained equations and time series forecasting of wind speed data. The study was motivated by the high prevalence of missing wind speed historic data. Findings based on the fully conditional specification under multiple imputations by chained equations, provided reliable wind speed missing data imputations. Further, the forecasting model shows, the smoothing parameter, alpha (0.014) close to zero, confirming that recent past observations are more suitable for use to forecast wind speeds. The maximum decadal wind speed for Entebbe International Airport was estimated to be 17.6 metres per second at a 0.05 level of significance with a bound on the error of estimation of 10.8 metres per second. The large bound on the error of estimations confirms the dynamic tendencies of wind speed at the airport under study.

## Introduction

Wind speed studies have increased in the recent past (Li and Shi 2010; Lun and Lam 2000). The interest by researchers may be attributed to the increasing magnitude and frequency of both positive and adverse effects caused by wind speeds (Dorvlo 2002; Calif et al. 2011). It has become apparent that there is a close correlation with the climate change phenomenon. Winds are known to be advantageous in some circumstances that include provision of wind energy; hence the desire for more regular big magnitudes of wind speeds (Qin et al. 2011). Strong wind speeds, however, can result in hazardous phenomena such as wind shears, an atmospheric phenomenon consisting of a sudden variation in intensity and direction of the wind. Wind shears are responsible for many incidents and accidents that occur during takeoff or landing and during low altitude flights. A venture into understanding the tendencies of their occurrence can improve control, management and planning of air traffic flow at an airport (Wesonga et al. 2013). Conversely, forecasting of winds, however, helps the wind energy planning and production sustainability; as well as the pilots and operators responsible for air traffic control in their ongoing surveillance activities thereby giving a real time warning of wind shear phenomena.

This process of wind forecasting provides much needed information for energy generation to complement hydroelectricity that is sometimes unreliable. In many African airports, however, wind data is scanty and where attempts to record them are made, they are not consistent over desired long periods of time to provide for more systematic statistical analysis (van Buuren 2011). For example, at Entebbe International Airport, although wind data were observed, recorded and used in developing daily forecasts, the capacity to maintain wind databases for a long time was curtailed by a lack of the necessary infrastructure and equipment. None of the reviewed literature on wind speed attempted to logically handle missing wind speed imputation. The main objective of this study was to perform missing data imputation on the available historic wind speed data at Entebbe International Airport. I also developed a wind speed time series model using the imputed data to provide knowledge about the behavior of wind speed in the absence of properly maintained actual databases for both wind speed and direction (Wesonga and Nabugoomu 2014). The long term goal is to use wind speed data to inform operations such as air traffic flow management and wind energy production.

### Data description and methodology

Daily wind speed data for the period 1995 through 2008 were collected from the Department of Meteorology. These data were transformed into decadal values to induce farther levels of presence of randomness of data that would promote statistical forecasting models. Given that wind energy is a function of maximum wind speed, the wind speed data were categorised into decadal wind speed dataset and a maximum value selected for each period. The tendency to focus only on the maximum wind speed was based on the fact that it is the category that is most likely to result in wind energy generation. Accidents and most incidences by aircrafts at airports are often caused by maximum wind speeds. It was, however, observed that some days, months and even years in the time period lacked records of wind speed. This incompleteness was the main focus of this study, thus the reconstruction of a prototype solution through employing multiple imputation methodology. Missing or incomplete data is an area of statistics that is challenging. The complexity of missing data phenomena is revealed in many studies that decide to drop variables with missing data. This study shows that such data can still be useful, as shall be demonstrated. Imputations when carefully done will mirror the actual data scenario. To achieve this, problem assessment and prototyping using R statistical computing program were carried out on the available wind speed data. Table 1 shows variables for this study.

**Table 1:** **Wind speed decadal data variable description**

**Variable name**	**Variable description**
Year	Year
Month	Month of the year
dmax1	Decadal one maximum wind speed
dmax2	Decadal two maximum wind speed
dmax3	Decadal three maximum wind speed

Daily data were collected for the period 1995 through 2008. These data were converted into decadal wind speed data values that represent maximum wind speed experienced over a period of ten days. The idea was to create randomness of missing wind speed data so that issues of bias within the data are eliminated. We also considered the fact that it does not require consistence of occurrence of maximum wind speeds for the wind speed to be harnessed for wind energy generation. Table 2 shows a sample of wind speed dataset where NA implies missing or incomplete wind speed data. After wind speed data imputations and testing, stochastic time series additive model was developed and wind speed predictions made as presented subsequently in this paper.

**Table 2:** **Sample of original maximum wind speed decadal data**

**Year**	**Month**	**dmax1**	**dmax2**	**dmax3**
2003	10	NA	NA	NA
2003	11	NA	NA	NA
2003	12	NA	NA	NA
2004	1	48	15	25
2004	2	20	20	13
2004	3	15	24	20
2004	4	22	25	20
2004	5	15	14	20
2004	6	15	12	15
2004	7	NA	NA	NA
2004	8	16	15	18
2004	9	18	10	15
2004	10	15	15	25
2004	11	15	16	18
2004	12	14	25	20
2005	1	NA	NA	NA
2005	2	NA	NA	NA
2005	3	NA	NA	NA
2005	4	NA	NA	NA
2005	5	NA	NA	NA

*NA means Missing Wind Speed Data.*

### Markov Chain Monte Carlo (MCMC) method

The Markov Chain Monte Carlo (MCMC) has its origin in physics as a tool for exploring equilibrium distributions of interacting molecules (Walsh 2004). However, in statistical applications, it is used to generate pseudorandom draws from multidimensional and otherwise intractable probability distributions via Markov chains. A Markov chain is a sequence of random variables in which the distribution of each element depends on the value of the previous one.

In MCMC, one constructs a Markov chain long enough for the distribution of the elements to stabilize to a common distribution. This stationary distribution is the distribution of interest. By repeatedly simulating steps of the chain, it simulates draws from the distribution of interest (Schafer 1999; Schafer 2003). In Bayesian inference, information about unknown parameters is expressed in the form of a posterior probability distribution. MCMC has been applied as a method for exploring posterior distributions in Bayesian inference. That is, through MCMC, one can simulate the entire joint posterior distribution of the unknown quantities and obtain simulation based estimates of posterior parameters that are of interest. Assuming that the data are from a multivariate normal distribution, data augmentation is applied to Bayesian inference with missing data by repeating the following steps:

1. *The imputation, I-step:*
  With the estimated mean vector and covariance matrix, the I-step simulates the missing values for each observation independently. That is, if you denote the variables with missing values for observation *i* by *Y*_*i(mis)*_ and the variables with observed values by *Y*_*i(obs)*_, then the I-step draws values for *Y*_*i(mis)*_ from a conditional distribution *Y*_*i(mis)*_ given *Y*_*i(obs)*_.
2. *The posterior, P-step:*
  The P-step simulates the posterior population mean vector and covariance matrix from the complete sample estimates. These new estimates are then used in the I-step. Without prior information about the parameters, a non-informative prior is used. You can also use other informative priors. For example, a prior information about the covariance matrix may be helpful to stabilize the inference about the mean vector for a near singular covariance matrix. The two steps are iterated long enough for the results to be reliable for a multiple imputed data set (Schafer and Olsen 1998). The goal is for the convergence of the iterates to their stationary distribution and then to simulate an approximately independent draw of the missing values.
  That is, with a current parameter estimate *θ*^(t)^ at *t*^*th*^ iteration, the I-step draws ${Y}_{mis}^{\left(t+1\right)}$ from *p*(*Y*_*mis*_/*Y*_*obs*_, *θ*^(*t*)^) and the P-step draws *θ*^(*t*+1)^ from $p\left(\theta /{Y}_{obs},{Y}_{mis}^{\left(t+1\right)}\right)$. This creates a Markov chain $\left({Y}_{mis}^{(1)},{\theta}^1\right),\ \left({Y}_{mis}^{(2)},{\theta}^2\right), \dots$ which then converges to the distribution *p*(*Y*_*mis*_, *θ*/*Y*_*obs*_).
  Table 3 presents a summary of the modules and functions used for performing the multiple imputations of the missing wind speed data and time series analysis. Further presentation and demonstration of the packages and R functions are well presented by the R development Team (R Core Team 2014).

**Table 3:** **Abridged R code for missing wind speed imputation and forecasting**

**Code line**	**R code for imputation and time series prediction of wind speed data**
*Code line 1*	# required R library functions
*Code line 2*	*library(VIM)*
*Code line 3*	*library(mice)*
*Code line 4*	*library(lattice)*
*Code line 5*	*library(“TTR”)*
*Code line 6*	*library(“forecast”)*
*Code line 7*	# Inspection of the missing data
*Code line 8*	*p <− md.pairs(wind)*
*Code line 9*	*marginplot()*
*Code line 10*	# MICE uses predictive mean matching, pmm
*Code line 11*	*imp <− mice(wind)*
*Code line 12*	# Further diagnostic checking
*Code line 13*	*imp$imp$values*
*Code line 14*	*c1 <− complete(imp)*
*Code line 15*	# Inspection of the distributions of original and the imputed data
*Code line 16*	*com <− complete(imp, “long”, inc=T)*
*Code line 17*	# Perform time series prediction modelling
*Code line 18*	*windts<− ts(c1$values,start=c(1995,1),frequency=36)*
*Code line 19*	# Decompose seasonal data
*Code line 20*	*windtscmpnts <− decompose(windts)*
*Code line 21*	*plot(windtscmpnts)*
*Code line 22*	# Seasonally Adjusting
*Code line 23*	*windtssadjusted <− windts - windtscmpnts$seasonal*
*Code line 24*	*windforecasts <− HoltWinters(windts, beta=FALSE, gamma=FALSE)*
*Code line 25*	*windforecasts$fitted*
*Code line 26*	*plot(windforecasts)*
*Code line 27*	*windforecasts$SSE*
*Code line 28*	# Forecast and forecast errors
*Code line 29*	*windforecasts1 <− forecast.HoltWinters(windforecasts, h=360)*

### Multiple imputations of wind speed data

The wind data has two main components; the wind direction, measured using the wind vane and wind speed, measured by an anemometer. Both of these parameters are important in understanding the behaviour of wind so as to make appropriate wind forecasting. However, for this study, only wind speed was considered because of its destructive strengths and effects which may or may not necessarily depend upon wind direction. Furthermore, given the daily records of wind speed, *10*-day category, referred to as decadal data, were formed from which maximum wind speed data point were extracted. Thus, decadal maximum wind speeds were derived using the following expression with *i* as decadal and *j* as day of the month within the given year;

$$ dma{x}_i= \max \left( windspeed\left(da{y}_j\right)\right);\ \mathrm{where}\ \mathrm{i}=1,2,3;\mathrm{j}=1,2,\dots, 10 $$

This process of data manipulation could not solve the problem of missing data as it was found out that several months, 47 out of a total of 168 (28%), of the data were missing. The problem was even bigger as in some cases maximum decadal wind speed data for two years (2002 and 2003) were missing.

In R, the *mice* package is known to impute incomplete multivariate data by fully conditional specification, FCS (Bartlett et al. 2014). Multivariate imputation by chained equations (MICE) first appeared in the year 2000 as an S-plus library and in 2001 as an R-Package. The first version introduced predictive selection, passive imputation and automatic pooling. Other extensions including imputing multi-level data, automatic predictor selection, data handling, post-processing imputed values, specialised pooling and model selection have since been advanced. The *mice* package advances two general approaches of imputing multivariate data based on MCMC. Firstly, the joint modelling (JM) that involves specifying multivariate distribution for the missing data and drawing imputations from their conditional distribution by MCMC techniques.

It should be noted that the basic idea of FCS is old and has been known by a number of names such as stochastic relaxation (Kennickell 1991), variable-by-variable imputation (Schafer 1999), regression switching (Van Buuren et al. 1999), sequential regressions (Raghunathan et al. 2001), ordered pseudo-Gibbs sampler (Heckerman et al. 2001), partially incompatible MCMC (Rubin 2003), iterated univariate imputation (Gelman 2004), chained equations (Van Buuren and Oudshoorn 1999) and fully conditional specification (Van Buuren 2007). The three main distinct multiple imputation inference steps by the FCS and adapted for this study are outlined below:

1. missing data are filled in *m* times to generate *m* complete data sets; a process known as imputation;
2. the *m* complete data sets are analysed by using standard procedures; commonly known as analysis and;
3. the results from the *m* complete data sets are combined for the inference; also known as pooling.

The original dataset presents 28% missing records of the 168. The MCMC approach iterates the given steps until they converge to their stationary distribution and then simulates an approximately independent draw of the missing wind speed values. MCMC was applied under the package *mice* and specifically using the FCS (fully conditional specification) routine in R language for statistical computing.

Three steps are employed under FCS as provided for in the package MICE; that is, imputation, analysis and pooling where functions; *mice()*, *with()* and *pool()* are applied respectively. At each step, storage classes are provided for; *mids*, *mira* and *mipo*. The final pooled dataset with completely filled missing data is then developed and stored in the database under the *mipo* class.

### Time series analysis of the imputed wind speed data

**Figure 1:**
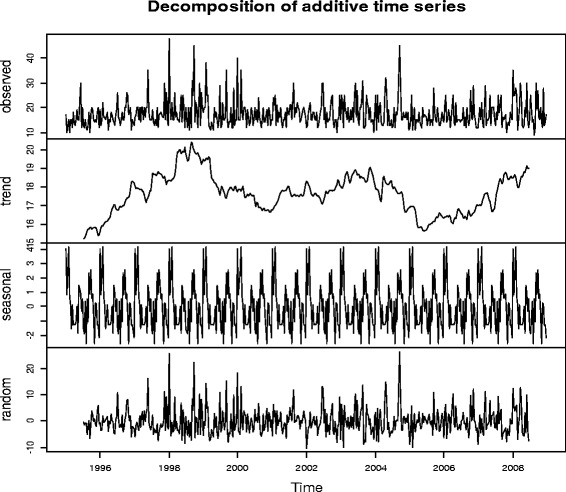
**Decomposition of additive time series for the imputed wind speeds.**

Further to the multiple imputations by chained equations (MICE) of the wind speed data at the Entebbe International Airport, time series analysis on the imputed data was performed. Figure 1 shows the additive time series plot showing the trend, seasonal and random components of the model besides the true observed plot, in this case, the imputed decadal wind speed data over the period 1995 through 2008. The purpose was to develop a wind speed time series model and use it to make forecasts.

**Figure 2:**
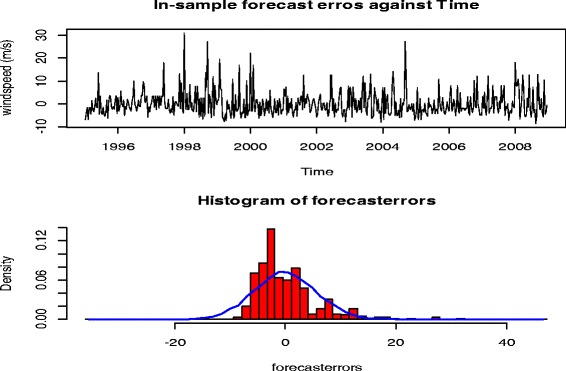
**Analysis of decadal wind speed forecast errors over the period 1995 – 2008.**

To check whether the forecast errors have constant variance, a time plot of the in-sample forecast errors was made. It is evident that the in-sample forecast errors approximated a mean at zero and a constant variance around the mean. The histogram plot of density against forecast errors is evident to this finding as shown in Figure 2.

Given the short-term time effects within wind speeds, forecasts were made with exponential smoothing described using an additive model with constant level and no seasonality assumptions, to make short-term forecasts. The additive Holt-Winters prediction function was employed to develop a wind speed forecasting model. The smoothing scheme begins by setting ${\mathrm{S}}_2$ to ${\mathrm{y}}_1$ where ${\mathrm{S}}_{\mathrm{i}}$ stands for smoothed observation or EWMA (exponentially weighted moving average) and y stand for the original observation. The subscripts refer to the time periods 1,2,..,n. Thus, S_t_ = αy_t_ + (1 − α)S_t − 1_ where, the smoothing parameter α is described as 0 < α ≤ 1; *t* ≥ 3. The speed at which older wind speed values are dampened or smoothed is a function of the smoothing constant α. When the smoothing parameter is 0, it implies that the current wind speed depend only on its speed one period before. When the smoothing parameter is 1, the wind speed will depend only on the current values. Determining the smoothing constant has been a challenge; (Hyndman and Khandakar 2007; Collopy and Armstrong 1992; Gardner and McKenzie 1985) agree that alpha can best be estimated from the data than just guessing.

**Figure 3:**
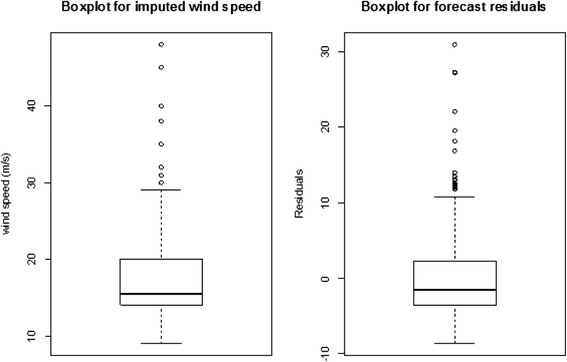
**Boxplots of the imputed wind speed and forecast additive model residuals.**

Figure 3 shows that the residuals of the exponentially smoothed time series model are also positively skewed demonstrating that the wind speed data series are also positively skewed.

## Discussions

Discussions are based on descriptive analysis for missing wind speed data, time series analysis for imputed and missing time series data.

### Descriptive analysis for level of missing wind speed data

The descriptive analysis shows that there were 168 records, an equivalence of the number of month between 1995 through 2008 considered. For each of the three decadal wind speeds studied, there were equal number of observations (121) and missing or incomplete observations (47). Correspondingly, the median year of observed wind speeds (2000) was the same for the three wind speed decadal data as it was the case for the missing or incomplete observations (2003). Outlier *missingness* in the data was observed in the earlier periods of 1995 and 1997 respectively for the three wind speed decadal data.

### Time series analysis of imputed versus original wind speed data

**Figure 4:**
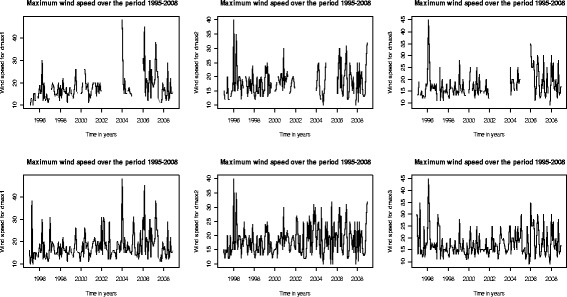
**Time series comparison between original and imputed wind speed.**

Figure 4 shows the level of truncation of decadal wind speed before multiple imputations by chained equations. As pointed out earlier, the proportion of missing wind speed data was about 28% and gaps within the time series show its exact distribution. It was evident that for two years between the years 2002 and 2004, there were no wind speed data; not because it was not collected, but probably because it was not kept in the data archives at the time of this study, hence missing. The imputed wind speed data in the corresponding decadal for the same period show a good fit having preserved not only the relations in the wind speed data, but also the uncertainties about these relations over time period.

### Differences between the imputed and original wind speed data

Wind speed data imputation necessitated scientifically imputing data gaps that may be at the beginning, within or at the end of the wind speed dataset. The power and accuracy levels of the imputation methods often vary and therefore, a survey of the most suitable approach was carried out to ensure validity of an imputed wind speed dataset. Although sometimes, imputation methods are capable of predicting future occurrences, necessary assumptions should be made to produce reliable results.

Whereas data characterisation may be a good approach to understanding the general structure of data, it does not offer a good solution where further data analysis is required to guide decisions and policy for national operations. Maintenance of character of data, however, is a key factor in determining the reliability of the imputation methods applied.

One of the standards of assessing imputation methods is the ability to preserve the structure and probability functions of the imputed data. Thus, an attempt was made to test the hypothesis that the hypothesis that there was a significant difference between the original wind speed and the imputed datasets *H*_*A*_ : *μ*_*original*_ ≠ *μ*_*imputed*_. Findings, using the T-test statistics (*t* = 0.3915; *P*(|*T*| > |*t*|) = 0.6955) showed that there was no significant difference between the original and imputed wind speed datasets. Thereby failing to reject the null hypothesis that presupposed mean wind speed of the original dataset was equal to the mean wind speed of the imputed wind speed dataset. These findings confirm the high level of reliability for the imputation method applied in this study.

### Time series model for the imputed wind speed data

**Figure 5:**
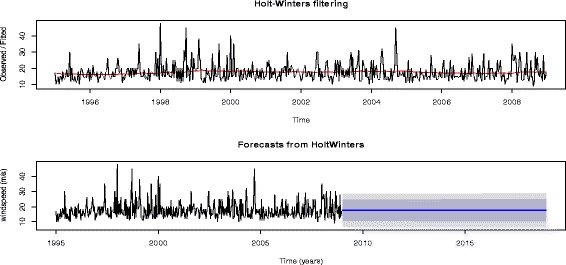
**Wind speed forecasting based on the imputed data.**

The alpha ($\upalpha =0.014$) computed from the data is relatively close to zero, implying that the forecasts are based on both recent and less recent observations (although somewhat more weight is placed on recent observations). Furthermore, this value implies that at rate of dampening is slow, hence confirming that recent observations are more relevant to make forecasts for decadal wind speeds. The wind speed forecasts as presented in Figure 5 show the plot for Forecasts from Holt-Winters are estimated at both 80% and 95% confidence intervals, shown by the lighter and darker parts of the plot respectively.

## Conclusions

In this study, missing decadal wind speed were imputed using multivariate imputation methods. The multiple imputation by chained equations (MICE), whose implementation in the R language for statistical computing was applied. The approach was found to preserve not only the relations and categorisations within the data, but also the uncertainties about these relations over time period. Furthermore, time series model for the imputed decadal wind speeds was developed with a view of presenting a simple exponential smoothing prediction model. Further works on time series modelling is recommended to reconstruct the stochastic tendencies of wind speeds at the case study. The maximum decadal wind speed for Entebbe International Airport was estimated to vary within (17.6 ± 10.8) metres per second.

Like all developing countries, wind speed data management, analysis and prediction is an area that receives the least priority given the competing demands these countries are faced with. Management of these vital data can be improved to minimise any threats to lives and property, especially during departures and arrivals (Wesonga et al. 2012). Technologically, low level wind shear alert systems (LLWAS) are recommended for management, analysis and predictions of wind phenomena.

On the positive side, further analysis and comprehension of the behaviour of these high wind speeds could be a good source of energy when harnessed by competent authorities in charge of energy generation. The wind energy would complement the energy demands for units of operations at the airport (Burton et al. 2011; Giebel et al. 2011). Data is a conveyor of information, complete and timely data, especially wind speed data at an international airport, if appropriately tapped can facilitate smooth airport operations.
